# Seasonal and Ecological Determinants of Wild Boar Rooting on Priority Protected Grasslands

**DOI:** 10.1007/s00267-024-01952-y

**Published:** 2024-03-14

**Authors:** Martina Calosi, Chiara Gabbrielli, Lorenzo Lazzeri, Niccolò Fattorini, Gloria Cesaretti, Lucia Burrini, Ottavio Petrillo, Francesco Ferretti

**Affiliations:** 1 Research Unit of Behavioural Ecology, Ethology and Wildlife Management—Department of Life Sciences—University of Siena, Via P.A. Mattioli 4, 53100 Siena, Italy; 2 NBFC, National Biodiversity Future Center, Palermo, 90133 Italy

**Keywords:** Foraging activity, habitat conservation, habitat-wildlife relationships, soil erosion, *Sus scrofa*, Wild pigs

## Abstract

Wild ungulates can influence various trophic levels, regulating carnivore abundance and affecting habitat structure. Conservation problems can arise when high ungulate densities threaten species or habitats with conservation concern. Assessing factors influencing the intensity of their impact is important to identify appropriate measures enhancing habitat conservation. We assessed factors influencing wild boar *Sus scrofa* pressure on EU protected grasslands in three protected areas of central Italy, by modelling the effects of environmental variables and wild boar density on rooting activity. We seasonally estimated rooting in 126 sampling plots from spring 2019 to spring 2021, and we used faeces counts to estimate summer wild boar densities. Estimates of density and rooting varied from 3.5 to 22.2 individuals/km^2^ and from 1.1 to 19.2%, respectively. We detected a clear seasonal trend in rooting activity, that peaked in autumn and winter. We also found a strongly positive correlation between spring-summer rooting and summer density, across sites. Rooting intensity was negatively related to the local extent of rock cover and increased with the 1 month-cumulative rainfall, the perimeter of the grassland patch, and the forest cover around plots. These results emphasise the tendency of wild boar to exploit feeding sites in ecotonal areas, i.e., at the interface between forest and meadows, which maximises security and ease of finding food resources. Actions aiming at the protection of focal plants in grassland habitats, as well as reducing wild boar presence, are supported (e.g. fencing and/or targeting population control at vulnerable patches).

## Introduction

Wild ungulates are key components of biological communities and can influence biodiversity through top-down cascading effects across trophic levels, as well as by acting as bottom-up regulators of carnivore abundance (Hebblewhite et al. 2005; Ripple et al. 2015). Under some conditions, wild ungulates can reach high densities, influencing plant and animal communities, and potentially affecting habitat structure (Côté et al. 2004; Barrios-Garcia and Ballari 2012; Foster, Barton, and Lindenmayer 2014; Barasona et al. 2021). Studies have shown both increases and decreases in plant diversity (Brunet et al. 2016) depending on plant species (Palacio et al. 2013) and type, intensity, frequency and extension of ungulate exploitation (Augustine and McNaughton 1998; Olaff and Ritchie 1998; Burrascano et al. 2015; Horčičková et al. 2019). Conservation problems can arise when high herbivore densities threaten species with conservation concern (Côté et al. 2004; Barrios-Garcia and Ballari 2012).

The wild boar *Sus scrofa* is the most widespread wild ungulate in the world (Ballari and Barrios-García 2014). It can live in a wide range of environmental conditions (Singer 1981), showing a clear potential to modify habitats and biotic communities through its feeding activity, thus representing an ecosystem engineer (Massei and Genov 2004; Bueno et al. 2009; Wirthner et al. 2011). This suid is an opportunistic omnivore, able to adapt its diet to spatio-temporal variation of food availability (Schley and Roper 2003; Markov et al. 2019). In particular, its typical foraging activity by digging the soil through rooting (Ballari and Barrios-García 2014) can affect topsoil as they search for belowground food resources, such as plant rhizomes, bulbs and earthworms. They can reach depths of 5–15 cm (Horčičková et al. 2019), turning up areas of hundreds of hectares (Massei and Genov 2004; Bueno et al. 2009; Barrios-Garcia and Ballari 2012; Bueno and Jimenéz 2014). The extent of impacted areas may vary annually, seasonally, and among habitat types (Welander 2000). Variation in wild boar rooting intensity can affect soil properties such as nutrient availability (Bueno et al. 2011; Bueno et al. 2013; Palacio et al. 2013), moisture (Mohr, Cohnstaedt, and Topp 2005; Tierney and Cushman 2006; Bueno et al. 2013), bacterial community structure (Wirthner et al. 2011), and richness of seed‐bank species (Bueno et al. 2011). In fact, rooting has been used as an index of wild boar pressure on habitats (Hone 1995).

In natural and semi-natural habitats such as grasslands and ecotones, i.e. substantially open habitats with small patches of trees and shrubs surrounded by woodlands, wild boar can find adequate feeding opportunities and cover (Thurfjell et al. 2009). Grasslands often represent biodiversity hotspots (Habel et al. 2013), including habitats with great conservation relevance because of their suitability for a large number of plant and animal species (Feurdean et al. 2018), some of which are rare and protected (e.g. orchids and several invertebrate/small vertebrate species, see Olmeda et al. 2019). In the last century, factors associated with man-made changes in land use, e.g., land abandonment and the resulting undergrazing and shrub encroachment, overgrazing and management intensification promoting habitat degradation, or conversion into arable land and other activities reducing habitat cover and favouring habitat fragmentation, have been identified as threats to their conservation (Habel et al. 2013; Olmeda et al. 2019). Assessing potential factors that may alter those habitats should be a priority to prevent their irreversible degradation, especially in stressful conditions such as harsh climates or overexploitation by herbivores (Tong et al. 2004; Chen et al. 2014; Fang and Wu 2022). Mediterranean grasslands appear to be particularly endangered because they have to face significant seasonal variations in weather patterns, ranging from near-drought to rainy periods. Moreover, they are subject to a recent, sharp increase in wild boar numbers (Massei et al. 2015), exposing these habitats to overexploitation especially through rooting and trampling, that may further endanger these ecosystems (Bueno et al. 2011; Barrios-Garcia and Ballari 2012). The attractiveness of these habitats may expose them to the risk of impacts which may negatively affect the conservation status of particular species, especially in case of high wild boar densities. However, information on the spatio-temporal variation of rooting intensity in Mediterranean grasslands across seasons, as well as its determinants, is scanty.

We considered wild boar rooting in grasslands belonging to priority habitats protected under the EU Habitats Directive (92/43/EEC), identified with the Natura 2000 codes 6210* “Semi-natural dry grasslands and scrubland facies on calcareous substrates (*Festuco-Brometalia*) important orchid sites*” and 6220 “Pseudo-steppe with grasses and annuals (*Thero-Brachypodietea*)”. They are key-habitats for many protected species (plants, birds, insects and other invertebrates, reptiles and mammals) and are considered a high priority for the conservation of wild pollinator species, such as butterflies, wild bees or hoverflies, as well as for other rare or protected animal and plant species. They also provide multiple benefits and ecosystem services, including carbon storage and prevention of soil erosion (San Miguel 2008; Olmeda et al. 2019). These semi-natural dry grasslands are one of the most endangered ecosystems in the world due to their dependency on land use history (San Miguel 2008; Olmeda et al. 2019; Labadessa et al. 2023). While moderate herbivory may be beneficial for grassland maintenance (Tälle et al. 2016), spatio-temporally concentrated impacts such as intensive wild boar rooting are expected to be detrimental, reducing plant cover (Singer et al. 1984), plant diversity (Singer et al. 1984; Hone 2002, Barrios-Garcia and Ballari 2012) and plant regeneration (Bueno et al. 2011; Barrios-Garcia and Ballari 2012), potentially exacerbating and speeding up the decline of grasslands.

Our objective was to evaluate the role of some environmental factors potentially influencing wild boar rooting in grasslands. Although rooting may be expected to be influenced by wild boar density, information is contradictory across studies (positive relationship: Hone 2002; Sandom et al. 2013; non-significant relationship: Massei et al. 2018; Adams et al. 2019; Ferretti et al. 2021). While a greater number of wild boar is expected to lead to higher disturbance levels (e.g. by increasing trampling, grazing and rooting), the role of density may be mediated by other factors. For example, a reduced availability of alternative resources as well as ease of digging may increase the attractiveness of under-ground food for wild boar and thus their propensity to root (Choquenot and Ruscoe 2003; Adams et al. 2019). By working in three study areas characterized by a gradient of wild boar densities, we first concentrated on the effects of population density as a factor potentially influencing rooting intensity.

We also investigated the effects of seasonality and landscape configuration, to identify sites and times of the year more affected by rooting. Seasonal variation in the intensity of rooting may be expected to occur following temporal variation of local resource availability (negative relationship), which usually peaks in spring-summer, and humidity (positive relationship), that is usually higher in autumn-winter (Welander 2000; Amici et al. 2012). Grasslands are also vulnerable to impacts indirectly determined by wild boar attraction to other potential food resources such as earthworms, insect larvae and other invertebrates, which build up a substantial protein intake for this suid (Baubet et al. 2003, 2004; Bueno and Jiménez 2014) and that are present in topsoil especially in late winter and early spring (Massei et al. 1996; Baubet et al. 2004; Cappa, Bani and Meriggi 2021). Moreover, wild boar have a marked flexibility in spatial ecology and tend to select forested habitats for protection (Kim, Cho and Choung 2019). This plasticity in habitat selection is expected to cause higher vulnerability to damage for open areas located close to forest edges (Thurfjell et al. 2009; Amici et al. 2012). We predicted that rooting activity (1) would peak in autumn and winter, due to the higher rainfall that makes moisture soil easier to dig (Hone 1995; Welander 2000; Sandom et al. 2013); (2) would be influenced by rainfall, that should affect the vegetation growth and soil softness; (3) would be negatively related to (3a) the extent of rock cover (Elledge et al. 2013; Ferretti et al. 2021), (3b) slope (Acevedo et al. 2011; Ferretti et al. 2021), and (3c) the extent of the patches of 6210/6220 habitat; (4) would be the greatest in the highest-density study area, and would show a positive correlation with estimates of wild boar density.

## Materials and Methods

### Study Areas

**Fig. 1:**
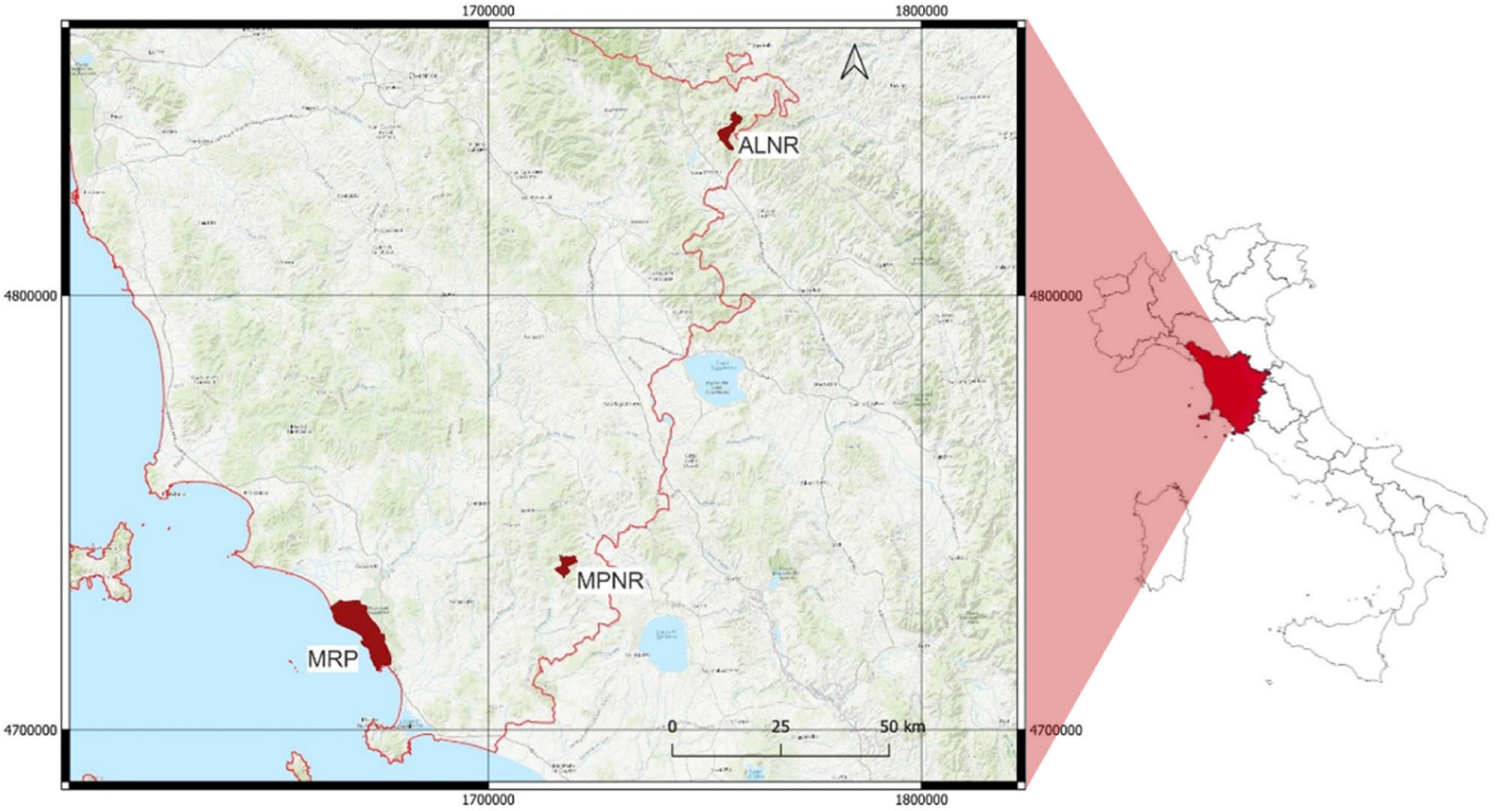
Location of our study areas: MRP (Maremma Regional Park), MPNR (Monte Penna Natural Reserve) and ALNR (Alpe della Luna Natural Reserve). The red line indicates regional borders

Our study was conducted in three protected areas in central Italy (Tuscany Region; Fig. 1): Maremma Regional Park (MRP), Monte Penna Natural Reserve (MPNR) and Alpe della Luna Natural Reserve (ALNR). These areas include relevant portions of grasslands classified with the Natura 2000 codes 6210* “Semi-natural dry grasslands and scrubland facies on calcareous substrates (*Festuco-Brometalia*) important orchid sites*” and 6220 “Pseudo-steppe with grasses and annuals (*Thero-Brachypodietea*)”.

MRP (maximum altitude of 417 m a.s.l.) is characterised by a typical Mediterranean climate. Most of the area is covered with Mediterranean sclerophyllic scrubwood (40%), with the presence of the oakwood component, mainly represented by the holm oak *Quercus ilex*, but also characterised by species such as *Juniperus* spp*., Myrtus communis* and *Phyllirea* spp. The northernmost sector includes a pinewood dominated by stone pine *Pinus pinea* (9%). The landscape is also covered by set-aside grasslands and cultivated fields (30%), wetland areas (5%) and other habitats (<1%). 13% of the park is covered by ecotones formed by abandoned olive groves—partially recolonised by scrubwood – and pastures, 5.5% of which is represented by habitat 6220 (Table 1). The remaining 2% is covered by human settlements.

**Table 1:** Summary of geographic and environmental features of the three study areas.

Study area	Location	Study area extent (km^2^)	6210/6220 habitat extent (code; km^2^)	N plots in 6210/6220 habitat	Mean elevation (m)	Mean Slope (%)	N plots for density estimation	Wild boar density gradient
ALNR	43.650348°N; 12.166402°E	15.4	(6210) 0.9	34	948	47	50	low
MRP	42.644144°N; 11.094017°E	89	(6220) 4.5	62	65	15.2	271	intermediate
MPNR	42.775285°N; 11.690544°E	11.1	(6210) 0.7	30	864	33.5	75	high

Location is given as mean latitude and longitude of sampling plots (WGS84)

The reported number of sampling plots in 6210/6220 habitat is that used to estimate wild boar rooting in habitat patches. The reported number of sampling plots for density estimation is that used to estimate wild boar densities in each whole study area, allowing a classification into three density gradients

*MRP* Maremma Regional Park, *ALNR* Alpe della Luna Natural Reserve, *MPNR* Monte Penna Natural Reserve

MPNR (maximum altitude of 1106 m a.s.l.) is largely characterised by calcareous hills and by caves and karst phenomena such as underground systems and sinkholes. The landscape is dominated by a mosaic of forests alternating with pastures, clearings, and rocky outcrops (Frignani et al. 2008). There is a prevalence of deciduous forests with *Quercus* spp., beech *Fagus sylvatica* trees and shrubs (82%). Meadows and grasslands growing on calcareous substrate make up 11.2% of the total area, and 56.1% of them are characterised by priority habitat 6210 (Table 1). Human settlements and cultivated areas represent 1.1% and 5.6% of the landscape, respectively.

ALNR (maximum altitude of 1453 m a.s.l.) is located on the Apennine ridge. Forests dominate the landscape, with associations of European beech *Fagus sylvatica* and Turkey oak *Quercus cerris*, as well as mixed woods mainly composed of Turkey oak and hophornbeam *Ostrya carpinifolia* (86.3%) (Viciani et al. 2013). Abandoned cultivated fields and grasslands also occur (10%) with 58% of these habitats being characterized by priority habitat 6210 with *Festuco-brometalia* facies and a rich population of orchids (Table 1). The remaining landscape is covered by anthropic settlements (0.9%) and cultivated areas (2.8%).

These areas host populations of wild boar along a gradient of density ranging from low (ALNR) through intermediate (MRP), to high (MPNR), as shown by previous studies (Fattorini and Ferretti 2020; Ferretti et al 2021; see Results). Other ungulates occurring in these areas are roe deer *Capreolus capreolus*, fallow deer *Dama dama* (not present in MPNR) and red deer *Cervus elaphus* (present only in MPNR). Wild boar predators are wolf *Canis lupus* and fox *Vulpes vulpes* (the latter on newborn offspring only).

### Rooting Estimates

From June 2019 to June 2021, we seasonally estimated wild boar rooting in grassland habitat patches using a plot-based approach. We used tessellation stratified sampling (TSS: Barabesi and Franceschi 2011; Barabesi et al. 2012): a grid (cell size: 150 × 150 m for MPNR and ALNR; 250 × 250 m for MRP) was superimposed on each whole study area and a 5-m radius plot was randomly placed within each grid cell. For the estimation of rooting, in accordance with the protocols widely adopted in environmental and forest surveys (e.g. Tomppo et al. 2010; Fattorini 2015), we only used plots that were located in habitats 6210 (ALNR; MPNR) or 6220 (MRP), discarding those falling outside, obtaining a total effort of 126 sampling plots (ALNR: 34, MPNR: 30; MRP: 62). We assigned geographic coordinates to the centre of each plot using QGIS 2.18, to allow their localization in the field through a portable Global Positioning System. To evaluate the possible effects of the season on the rooting activity of wild boar, these surveys were repeated seasonally on the same plots for all three study areas, in late February-March (mid-April only in MPNR in 2021), June-early July, September, and December, as representative for rooting activity occurred during the previous season (i.e. winter, spring, summer and autumn, respectively). Once in the field, plots were marked with pickets and tape to allow their detection in following surveys. In each plot, we used a 5-m rope to identify the effective area to be considered for visual estimates of rooting. For each plot, the percentage of ground with rooting was therefore visually estimated. Following previous studies, the rooting percentage was recorded in classes (0; present but lower than 1%; 1–5%; 6–10%; 11–15%; etc.; Fattorini and Ferretti 2020; Ferretti et al. 2021), considering the median value of each class for analyses (0; 0.5%; 3%; 8%; 13%; etc). The percentage of rock cover in each plot was also visually estimated according to the same evaluation scale (Ferretti et al. 2021).

### Faeces Counts for Density Estimates

Consistently with previous studies conducted in the same areas, we estimated wild boar densities related to each whole protected area through faecal counts (Fattorini et al. 2011; Ferretti et al. 2016; Fattorini and Ferretti 2020; Ferretti et al. 2021). We used tessellation stratified sampling (TSS: Barabesi and Franceschi 2011; Barabesi et al. 2012), with 5-m radius circular plots placed within spatial units partitioning the study area. For the two smaller study areas (MPNR and ALNR) we partitioned each area into polygons of equal size and a plot was randomly placed within each of them (Ferretti et al. 2021). Based on previous studies (Fattorini et al. 2011; Ferretti et al. 2016; Fattorini and Ferretti 2020; Ferretti et al. 2023), we adopted a two-stage sampling strategy for our largest study area (MRP), in which we stratified the area according to main habitat/land cover, and local features. We identified 13 strata (two strata with scrub, three strata with pine and marshland, two strata with ecotones and olive groves, six strata with cultivated fields) and allocated plots proportionally to strata size. In the largest strata (i.e., scrub north and south, and a pine stratum) a two‐phase strategy was adopted: strata were partitioned into spatial units according to natural or artificial bounds (e.g., lanes, streams, cultivation bounds), then a sample of them was selected and was divided into polygons of equal size, where we randomly placed the plots (one plot per polygon). In the other strata, a one‐phase TSS strategy was used, with plots directly and randomly placed within each polygon (one plot per polygon, Fattorini et al. 2011). Overall, we used a sampling effort of ~1 plot/0.3 km^2^, for a total of 396 sampling plots.

We used the faecal accumulation rate technique (Mayle et al. 1999). This method involves visiting the same plot twice: a first survey that requires clearing the plot from all the wild boar faeces, which can be identified from those of other ungulates by their unequivocal shape and size; a second survey, conducted 35–40 days later, based on decay of boar faeces (Massei et al. 1998), to count all wild boar faeces accumulated in the plot since the clearance day (Mayle et al. 1999). We assigned geographic coordinates with the same method implemented for rooting plots, as explained in the relevant paragraph, to find the plots through a portable Global Positioning System unit. We performed the clearing survey from mid-June to early July, each year (2019, 2020, and 2021). To limit the potential subjectivity in faeces identification, each year the same operator carried out the faeces count in the same plots for both the clearance and the counting passages. To help localizing plots, we marked the centre of each plot with red and white tape. Wild boar densities were derived by using the daily defecation rate (DDR) estimated by Fattorini and Ferretti (2020) on a semi-captive wild boar population ~40–110 km from our study areas, in summer (6.7 faeces/individual/day). Methodological details and theoretical justifications for density estimation were given in Fattorini et al. (2011) and Fattorini and Ferretti (2020), where an unbiased estimator of faeces abundance and a conservative estimator of its standard error have been provided.

### Environmental Predictors

We calculated the cumulative rainfall which occurred in the month and the three months preceding surveys, in each study area, by using the sum of daily rainfall recorded by the meteorological station closest to each study area (Servizio Idrologico Regione Toscana: Alberese station for MRP and Semproniano station for MPNR; DEXT3R Emilia-Romagna: Badia Tedalda station for ALNR). At the global scale, the effect of accumulated rainfall on vegetational productivity ranges from 0.6 to 2.8 months depending on vegetation type (Ding et al. 2020). Although it represents a proxy, we decided to consider rainfall accumulated in one month as effective on grasslands and open shrublands, i.e. the main vegetation types near our sampling areas, and three months accumulated rainfall as the most meaningful for all vegetation types present throughout the study areas (Ding et al. 2020). We derived the slope (%) of each plot using the software QGIS 3.28 and the digital elevation model provided by Tuscany Region database (http://dati.toscana.it; resolution: 10 × 10 m). We used the land cover layer provided by SITA: cartoteca Regione Toscana (hereafter SITA; https://www502.regione.toscana.it) and the QGIS buffer feature to calculate the percentage of woodland in a circular buffer with radius of 1100 m around each plot located in grassland habitat 6210/6220. The buffer size was determined as the mean of yearly home ranges (weighted by number of individuals sampled) obtained in study areas close and ecologically comparable to ours (Boitani et al. 1994: Colline Metallifere – Siena Province; Massei et al. 1997: MRP). We obtained the average home range for females (3.9 km^2^ – radius: 1100 m) and males (7.1 km^2^ – radius: 1500 m). Since it is impossible to identify the individuals responsible for rooting activity (in particular their age class and sex), we decided to perform the analyses using the smaller radius, to be more conservative in including the maximal area likely used by the majority of the wild boar population. We calculated the extent and perimeter of 6210/6220 habitat patches through QGIS and the protected habitats layer provided by SITA. Although it represents a proxy to analyse the effect of the 6210/6220 habitat extension, as the patch perimeter was strongly related to the patch area (*r* = 0.87; *p* < 0.001), we used the former for analysis, as the latter was collinear to the forest cover percentage in the buffer (*r* = −0.74). We used the pedological database provided by SITA to identify the soil depth classes in the protected habitats patches. This database provides a six-class classification of soils based on the depth useful for roots growth (A1: >100 cm; B2: 75–100 cm; C3: 50–75 cm; D4: 25–50 cm; G7: 10–25 cm; H8: <10 cm); in our study areas, only the classes A1, B2 and D4 were present. Since water availability may be an important limiting factor for wild boar (Massei et al. 1997), we considered the distance between plots and persistent water resources as a variable. Although ephemeral water bodies such as small ponds and ditches are not georeferenced, the habitats 6210 and 6220 are located on dry/well-drained soils with a small carbonate content (Olmeda et al. 2019). As water retention increase linearly with carbon content (Emerson 1995), we assumed that in our study areas the probability of creating surface waterlogging is low. Thus, persistent water resources are likely to be the main water resources available for wild boar. We merged three different layers (SITA: hydrographic network; water infrastructure layer; water bodies layer) to create a detailed map of persistent water sources. Then, we calculated the distance between plots and water resources using the QGIS NNJoin plugin.

### Statistical Analyses

We investigated factors affecting wild boar rooting using generalized linear mixed models (GLMMs; Zuur et al. 2009). Our response variable was the proportion of rooting assessed in each sampling plot, reflecting the impact on protected grasslands in every seasonal survey, throughout the study years. We modelled the rooting proportion using beta errors (logit link), as recommended when modelling continuous proportions. Thus, to enable the use of beta errors, we converted the percentage of rooting into relevant proportions within the range 0‒1 and transformed every 0 into 10^−6^ without altering its biological meaning (conversely, a proportion of rooting equal to 1 was never found). We tested whether our response variable varied according to predictors while accounting for repeated measures conducted in each sampling plot and study year as crossed random intercepts. As fixed effects, we included (1) study area (categorical; reference level: ALNR), (2) rock cover (continuous, as %), (3) season (categorical; reference level: spring), (4) soil depth class (categorical; reference level: A1 0–10 cm), (5) perimeter of 6210/6220 habitats patches (continuous, in metres), (6) woodland cover in a 1100 m buffer (continuous, as %), (7) distance from the nearest persistent water source (continuous, in metres), (8) slope (continuous, as %), (9) three months-cumulative rainfall (continuous, in millimetres), and (10) one month-cumulative rainfall (continuous, in millimetres). As we found high collinearity between the two scales of cumulative rainfall (*r* = 0.78), we decided to perform two different models, one considering each cumulative-rainfall scale. We considered the AICc value of each model (i.e., the Akaike Information Criterion corrected for small sample size) and we selected the model with the one month-cumulative rainfall variable as parameter, i.e. the one with the lower ΔAICc value (one month-cumulative rainfall ΔAICc = −10872.8; three months-cumulative rainfall ΔAICc = −10845). After this selection, we found no substantial multicollinearity among covariates (r < |0.6|) or predictors (VIF_mean_ = 2.31, VIF_min_ = 1.07, VIF_max_ = 5.8). Covariates were scaled to improve model convergence and to allow assessing the relative importance of each predictor.

Statistical analyses were conducted following the information-theoretic approach, by evaluating multiple competing a priori hypotheses (model selection; Harrison et al. 2018). The multi-model selection approach is particularly recommended whenever multiple hypotheses are plausible to assess the combination(s) of predictors that best contribute to support empirical data (Grueber et al. 2011; Harrison et al., 2018). Through this approach, competing models are ranked and those receiving the best support are selected based on the balance between their simplicity and goodness of fit (Aho et al. 2014). Once a subset of candidate models is obtained using such criterion, selected models can be used to predict the measured indicator by estimating the effects of predictors. Previous studies identified major predictors potentially influencing wild boar rooting (e.g. density: Fattorini and Ferretti 2020; rock cover: Ferretti et al. 2021; slope: Ferretti et al. 2021; distances to resources: Bueno et al. 2009). Therefore, we could not discard any combination of these explanatory variables in advance, as all the relevant underlying hypotheses could be meaningful biologically. Consequently, from the full model, we performed an all-subset model selection to rank and weight all possible models, each corresponding to the relevant combination of fixed effects. Each model evaluated, as well as the null, random intercept-only model, could in fact represent a different a priori hypothesis (Harrison et al. 2018). We considered the AICc value of each model and its difference with respect to the model with the lowest AICc value, i.e., ΔAICc. We were conservative by following the ‘nesting rule’, to avoid retaining overly complex models (Harrison et al. 2018): we did not select models with ΔAICc ≥ 2 than the best model (the model with the lowest AICc value), as well as models with an AICc value greater than that of any simpler alternative, achieving a set of top-ranked models. Model weight was standardized within the subset of selected models. Selected models are reported in Table S1. We estimated coefficients of predictors, 95% confidence intervals, and variance of random effects from the top-ranked, best model. The effects of predictors were assessed by checking whether 95% confidence intervals of coefficients overlapped 0. Best models were validated by checking residual patterns (Zuur et al. 2009). We carried out model selection and GLMMs through the R packages *MuMIn* (Bartoń 2012) and *glmmTMB* (Brooks et al. 2017), respectively.

To evaluate the relationship between estimates of rooting in 6210/6220 habitat patches and wild boar density in the whole protected area, we calculated linear correlation coefficients between estimates of rooting and densities. Since the wild boar is a seasonally breeding animal with an oestrus normally occurring in summer and early autumn (Mauget 1986), we tested the Pearson’s correlations between summer density estimates and the rooting activity in 6210/6220 habitats in both spring and summer (i.e., June and September). For this analysis, we did not consider autumn (i.e., estimates of rooting in December) and winter (i.e., rooting in late February-March/mid-April) because local variations of wild boar densities across seasons (e.g., because of delayed reproduction or movements towards protected areas from unprotected sites during the hunting season, occurring in October-January, Massei et al. 2011; Brogi et al. 2022) could make our summer estimates not representative of densities in the cold period. We first checked for the bivariate normality of data (Mardia’s multivariate tests for small samples-corrected skewness, z_1_, and kurtosis, z_2_; June: z_1_ = 3.207, df = 4, *p* = 0.524; z_2_ = −0.731 df = 4, *p* = 0.465; September: z_1_ = 1.420, df = 4, *p* = 0.841; z_2_ = −1.303 df = 4, *p* = 0.193). The Pearson’s correlations were calculated with R 4.3.1 (R Core Team 2023).

## Results

**Fig. 2:**
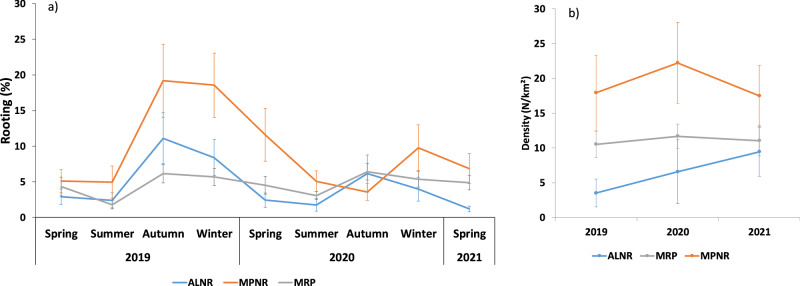
**a** Seasonal estimates of rooting (mean for each season ± SE) and (**b**) wild boar density estimated in summer (mean for each year ± SE), from 2019 to 2021 in each study area

The percentage of rooted areas ranged seasonally from 3.6 to 19.2% in MPNR, from 1.8 to 6.4% in MRP, and from 1.2 to 11.1% in ALNR (Fig. 2a). Wild boar density was the highest in MPNR, where it spanned 17.5–22.2 individuals/km^2^ (CV: 25–30%), intermediate in MRP (10.5–11.7 individuals/km^2^, CV: 15–20%), and the lowest in ALNR (3.5–9.5 individuals/km^2^, CV: 37–57%; Fig. 2b).

Three models for spatio-temporal variation of rooting were selected: all of them included the effects of season, study area, 1 month-cumulative rainfall, patch perimeter and forest cover percentage in the buffer; moreover, the best model included the effect of slope and rock cover, not included in the second-best (slope) and third-best (rock cover; Table S1).

**Fig. 3:**
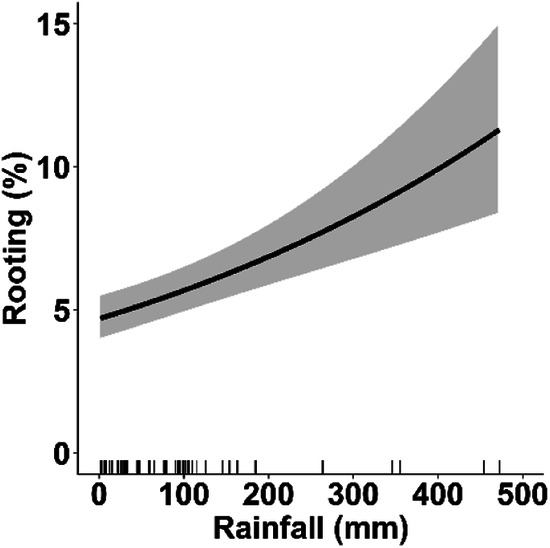
Effect of one month-cumulative rainfall on wild boar rooting, estimated by GLMMs. Prediction accounts for plot-repeated surveys and study year as random intercepts, showing the average effect across the three study areas. Marks along x-axis show the distribution of observed values for this covariate. Black lines: predicted values. Grey bands: 95% confidence intervals

**Fig. 4:**
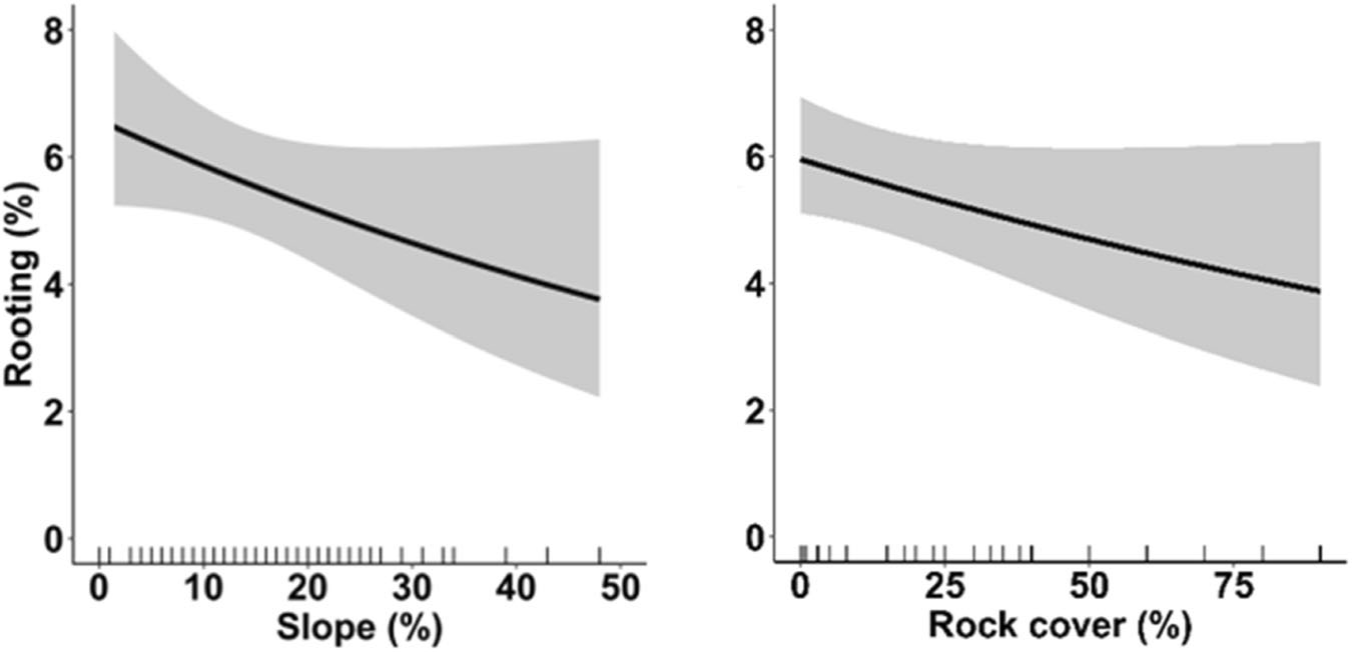
Effects of slope and rock cover on wild boar rooting, estimated by GLMMs. Predictions account for plot-repeated surveys and study year as random intercepts, showing the average effect across the three study areas. Marks along x-axis show the distribution of observed values for each covariate. Black lines: predicted values. Grey bands: 95% confidence intervals

**Fig. 5:**
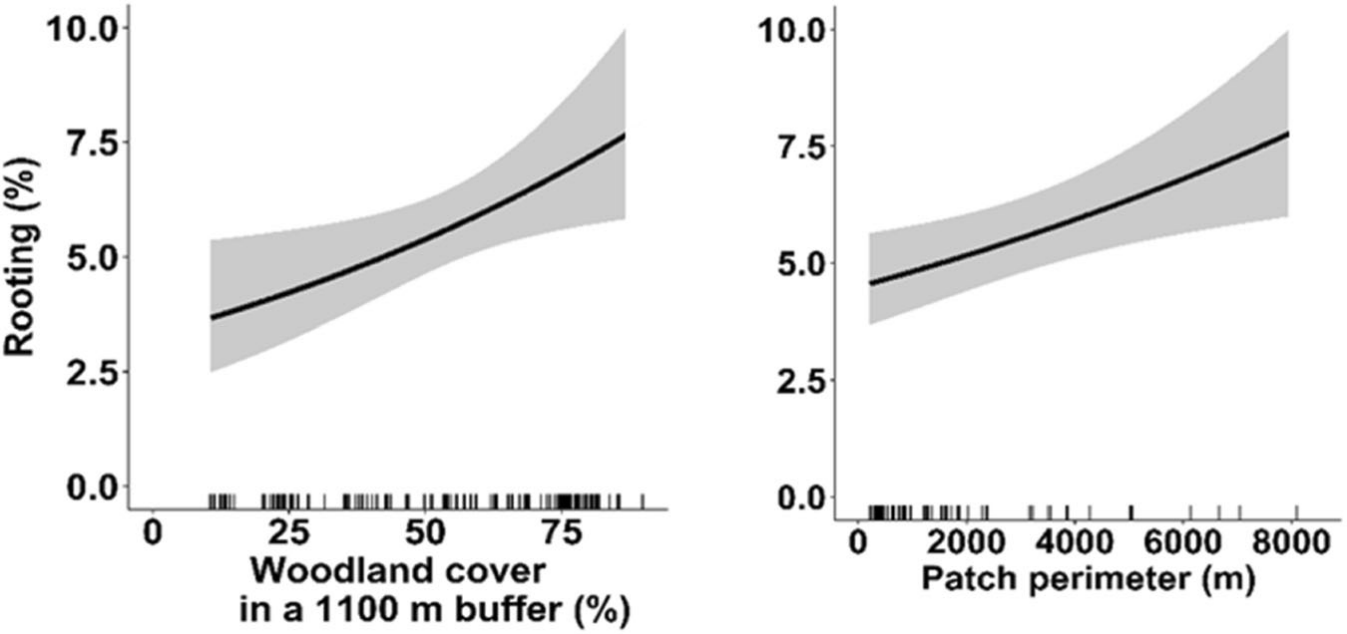
Effects of woodland cover in a 1100 m buffer and 6210/6220 habitat extent (approximated by patch perimeter) on wild boar rooting, estimated by GLMMs. Predictions account for plot-repeated surveys and study year as random intercepts, showing the average effect across the three study areas. Marks along x-axis show the distribution of observed values for each covariate. Black lines: predicted values. Grey bands: 95% confidence intervals

**Fig. 6:**
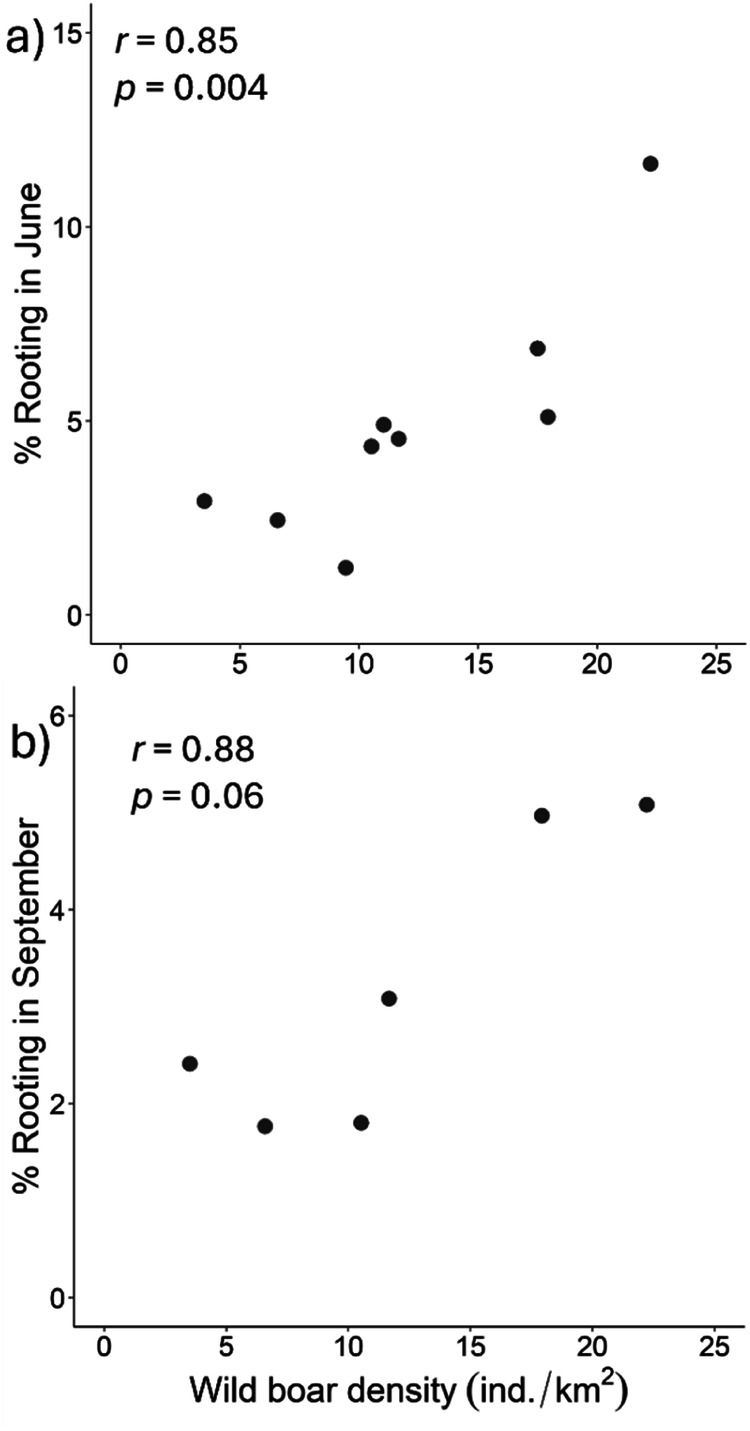
Correlation between wild boar density estimated in summer and rooting percentage in (**a**) June and (**b**) September

In accordance to our prediction (1), a seasonal trend of rooting was observed, with an increase in the percentage of rooted soil in autumn and winter in all study areas (Table 2). A subsequent decrease in the percentage of rooted soil was observed in the spring-summer period. Prediction (2) confirmed an increase in the rooting percentage with the cumulative rainfall in the previous month (Table 2; Fig. 3). Prediction (3) confirmed that the rooting intensity increased with decreasing rock cover and with decreasing slope, although the effect of the latter was weak, as its 95% CIs included ‘0’ (Table 2; Fig. 4). Moreover, rooting increased with increasing percentage of forest cover in the buffer and patch perimeter (Table 2; Fig. 5). In line with our prediction (4), rooting was the greatest in MPNR (Table 2; Fig. 2). Considering rooting surveys conducted in June and September, relevant estimates of rooting showed high and positive correlations with density estimates (June: *r* = 0.84, *p* = 0.004; September: *r* = 0.88, *p* = 0.06; Fig. 6).

**Table 2:** Parameters estimated from top-ranked GLMMs predicting wild boar proportion of rooted area in sampling plots: coefficients (B) and 95% confidence intervals (95% CIs)

Response variable	Predictor	B coefficient	95% CI
Proportion of rooting	Intercept	−3.303	−3.613; −2.993*
σ²_Year_ = <0.001	Slope (%)	−0.108	−0.245; 0.030
σ²_Plot_ = 0.236	Patch perimeter (m)	0.189	0.053; 0.324*
	Woodland % in a 1100 buffer	0.246	0.049; 0.443*
	Rock cover (%)	−0.103	−0.220; −0.015*
	1 month-cumulative rainfall	0.210	0.127; 0.293*
	Study area (MRP)	0.501	0.091; 0.911*
	Study area (MPNR)	0.954	0.602; 1.306*
	Season (summer)	−0.266	−0.434; −0.099*
	Season (autumn)	−0.025	0.231; 0.181*
	Season (winter)	0.301	0.128; 0.474*

Variance of random intercepts (σ^2^) is also shown. Reference category for ’Study area’ is ALNR. Reference category for ’Season’ is spring. An asterisk marks the coefficients whose 95% CIs do not include 0

## Discussion

There is a growing concern about the potential impacts of increasing densities of wild boar on the conservation status of vulnerable habitats (Massei and Genov 2004; Barrios-Garcia and Ballari 2012; Bengsen et al. 2014). Identifying key-factors influencing wild boar impacts is important to address specific management actions to mitigate the negative effects on habitats and species with conservation concern. We analysed factors influencing the spatio-temporal variation in indices of wild boar rooting activity on grasslands across three Mediterranean protected areas. We observed that rooting indices (i) peaked in autumn and winter, (ii) decreased with increasing rock cover, increased with the percentage of forest in a buffer around the site and with the cumulative rainfall in the previous month, and (iii) were positively associated with wild boar density.

Evolutionary processes have led individuals to adapt their behaviours to biotic and abiotic seasonal changes (Tauber and Tauber 1981; Mayr 1982). As a result, animal species have been shown to either modify their food habits to adapt to seasonal variations of availability of key resources, or to migrate, following the shifting distribution of trophic resources (Fryxell and Sinclair 1988). Our results reflected adaptive strategies of wild boar, consisting of seasonal dietary changes following variations in food availability (Herrero et al. 2006; Ballari and Barrios-García 2014; Laguna et al. 2021). Lower rooting indices were observed in spring and summer: in Mediterranean countries, these seasons are usually characterised by lower precipitation levels than colder seasons. Dryness is expected to increase ground hardness, which in turn should make the soil more difficult to dig. Availability of accessible and energetic alternative food resources may also limit wild boar use of grasslands: in late spring-summer, crops constitute a large proportion of the wild boar diet (Herrero et al. 2006), likely contributing to make natural/semi-natural grasslands less attractive for them. Conversely, autumn and winter are usually characterised by the highest levels of humidity and precipitation, making the soil easier to dig (Hone 1995; Welander 2000; Sandom et al. 2013), favouring the efficiency of wild boar olfactory sense (Brivio et al. 2017), and prompting an increase in rooting activity. Moreover, in autumn-winter, soils are richer in edaphic fauna due to arthropods surviving the harsh temperatures in a state of diapause underground (Tauber and Tauber 1981). These invertebrates represent a significant source of proteins for wild boar females, which have to face winter pregnancy (Schley and Roper 2003; Wilcox and Vuren 2009). Effects of cumulative rainfall variations on rooting were supported by our analyses, suggesting two possible, non-mutually exclusive effects of rainfall. An indirect effect of rainfall on rooting activity could occur by the increased rain-dependent soil and vegetation productivity, whereby wild boar may increase rooting due to higher availability of rain-mediated food resources. A direct effect of rainfall on rooting could also occur, as rain would make the soil softer to dig, thus favouring rooting activity. Potential effects of rainfall acting at shorter – and more immediate – temporal scales, e.g., in the previous days/weeks, may not be ruled out. Longer-term, multi-year studies would be needed to test for the site-specific effects of inter-annual variations of precipitation regimes on rooting. For example, in Grosseto province (where MPNR and MRP are located) 237.3 mm of rain were reported to occur in 20 days, in November 2019, whereas in November 2020 it rained 28.8 mm in 5 days, which might have influenced the lower rooting activity recorded in autumn 2020 than in autumn 2019 (data: Meteorological Service, Province of Grosseto). Understanding the relationships between rainfall and rooting deserves further investigation across multiple temporal scales.

Protected areas may serve as a refuge for wildlife during the hunting season (Grignolio et al. 2011; Santilli and Varuzza 2013; Colomer et al. 2021), which corresponds to October-January across the landscapes where our study areas were located. Thus, increased rooting in autumn-winter might be the result of a seasonal increase in wild boar use of protected areas to avoid hunting grounds. However, available information on the “reserve effect” is limited and contradictory, suggesting that its occurrence and magnitude are context-dependent (Grignolio et al. 2011; Santilli and Varuzza 2013; Brogi et al. 2020; Colomer et al. 2021). While our smallest study areas (MPNR and ALNR) are located in ecological continuity with neighbouring wooded areas, the MRP is bordered by the sea at its western side and is surrounded by agricultural and anthropized landscapes at the other sides. Thus, the potential for wild boar movements between protected areas and hunting grounds may differ across our study areas. Since seasonally-explicit density estimates through faeces counts could be heavily hampered during rainy seasons such as autumn/winter (due to accelerated faeces decay, in turn altering density estimations), data based on GPS telemetry would be needed to test for the potential of individual movements across the borders of protected areas in affecting the environmental impact of wild boar on grasslands during autumn-winter. Nevertheless, the implementation of population control within protected areas during periods of regular hunting in unprotected grounds may contribute to reduce wild boar pressure on protected habitats.

Although the regrowth of plants in rooted areas should be quantified, our results suggest the persistence of rooted ground at the end of the growing season of vegetation. Suggestively, preliminary work conducted in June in 1 × 1 m sample quadrats deployed on totally rooted patches of 6210/6220 grasslands, at the end of winter, in MRP and MPNR, suggested that *c*. 40–80% of the ground was still uncovered by vegetation (*n* = 90 quadrats in 2020–2021, our unpublished data), which would indicate that the short-term recovery of grassland was scarce and that rooting in autumn-winter could impose a significant reduction in space available for vegetation growth during the following vegetative season. However, the potential for medium-to-long-term vegetation recovery as well as for wild boar rooting to trigger shifts in the specific composition of plant communities should be assessed (Burrascano et al. 2015; Genov et al. 2017).

Our results provide new information on the debated relationship between rooting intensity and wild boar density. While several studies showed a positive relationship (Anderson and Stone 1993; Hone 2002; Sandom et al. 2013), others did not find support for higher rooting intensity in sites with greater density of wild boar (Massei et al. 2018; Adams et al. 2019; Ferretti et al. 2021). We showed that rooting peaked in the study area with the highest wild boar density, and provided strong support for a positive correlation between rooting intensity in spring and summer in 6210/6220 protected habitat and wild boar density. Availability of alternative resources and soil characteristics are key-factors influencing rooting activity (Baubet et al. 2004; Bueno et al. 2009; Lombardini et al. 2017), expectedly leading to habitat-specific and season-specific patterns. For example, grassland attractiveness may be favoured by rainy conditions, whereas it would be expected to decrease when alternative palatable resources are highly available (e.g., acorns and beechnuts). A previous work conducted in sampling plots across all habitat types in six protected areas (including also our three study areas) found scarce support for a correlation between rooting and wild boar density (Ferretti et al. 2021). Therefore, we suggest that the correlation between rooting and density may be better investigated by considering a habitat-specific approach such as that pursued in this study. Moreover we worked in relatively small protected areas (*c*. 10–100 km^2^), where processes acting at the study area scale may be expected to influence patterns observed at local scales (i.e., habitat patches). Conversely, at larger spatial scales, such as in larger protected areas, the relationship between overall population density and rooting intensity may be expected to be highly influenced by specific factors acting at smaller, local scales and influencing wild boar attraction to grasslands. Although autumn and winter densities of wild boar were not available, we found that rooting indices were the greatest in the high-density area also in these seasons, suggesting some degree of consistency in wild boar density.

Rooting indices increased with decreasing rock cover and, weakly, with slope steepness, confirming previous findings (Ferretti et al. 2021). Steep and rocky ground can reduce soil moisture, especially in priority-protected habitats 6210/6220 that are characterised by calcareous substrates, a condition that prevents water retention and favours rapid percolation, in turn accentuated by steepness (Hone 1995; Olmeda et al. 2019). Moreover, rooting indices increased with the percentage of nearby woodland cover and the perimeter of priority habitat patch size, i.e. the availability of forest-edges. These results are in line with other studies that showed a preference for marginal/ecotonal habitats by wild boar due to the coincident availability of both trophic resources and sheltering sites (Wilson 2004; Thurfjell et al. 2009; Amici et al. 2012; Fattorini and Ferretti 2020; Ferretti et al. 2021; Laguna et al. 2021). Frequently, *Thero-Brachipodietes* and *Festuco-Brometalia* facies host valuable and palatable species such as orchids and other herbaceous plants, which are expected to further increase wild boar attraction to grasslands, especially if they are located close to the woodland edge. Thus, our results confirm the tendency of wild boar to search for feeding sites close to forest, suggesting that patches with open habitats surrounded by woodland are the most vulnerable to rooting.

## Conclusions and Implications for Conservation

Within its native range, the wild boar has coevolved with local grasslands and native plants, suggesting that rooting pressure should not be considered as a threat to habitat conservation per se. For instance, a recent study has shown that rooting activity may locally stimulate favourable habitat conditions for some butterfly species (Labadessa and Ancillotto 2023). However, several anthropogenic factors have led to a modification in habitat cover, and influenced wild boar distribution and abundance (e.g., through artificial releases, supplemental feeding or by providing highly energetic food through agriculture) (Barrios-Garcia and Ballari 2012; Massei et al. 2015), potentially aggravating the negative consequences of wild boar activity for habitat conservation. Human activities such as landscape modifications, changes in land use and livestock management have influenced semi-natural dry grasslands and, nowadays, both the abandonment of traditional extensive management practices and their intensification have been identified as threats for the conservation of these habitats (San Miguel 2008; Olmeda et al. 2019). Main identified threats include, among the others, land abandonment and undergrazing, overgrazing, changes and/or intensification of management practices, or conversion into arable land or other activities reducing habitat cover (San Miguel 2008; Olmeda et al. 2019). The increase of wild boar densities at a continental scale may expose vulnerable environments such as grasslands and scrublands to an additive threat (Barrios-Garcia and Ballari 2012; Massei et al. 2015). By identifying key seasonal and ecological factors associated with rooting activity, as well as by providing support to a positive relationship between wild boar density and habitat-specific rooting pressure, our study offers insights about potential wild boar impact on priority protected grasslands. In particular, management actions would be supported to protect grassland patches on flat ground surrounded by forest habitats (e.g., through small fences preventing wild boar rooting on focal plants or plant groups, where/when feasible), as well as to limit wild boar presence/density and its attraction to grasslands, in sites and periods where/when wild boar rooting imposes a threat to vulnerable grasslands (e.g., through dissuasive and/or control methods, Cromsigt et al. 2013).

### Supplementary information

Supplementary material
