# Supplementary material for: Seasonal and Ecological Determinants of Wild Boar Rooting on Priority Protected Grasslands
Source: Environ Manage. 2024 Mar 14;74(2):268–81. doi: 10.1007/s00267-024-01952-y (PMC11227453; doi:10.1007/s00267-024-01952-y)
Supplement: Supplementary file 1 — Supplementary material [file 267_2024_1952_MOESM1_ESM.pdf]

Table S1. Result of model selection: best model and the other selected models with ΔAICc<2 while accounting for nesting, each with k (i.e., numbers of parameters), AICc value, ΔAICc and model weight.

**Model rank**	**Model**	**k**	**AICc**	**ΔAICc**	**Standardized weight**
Best	Patch perimeter + Woodland percentage in a 1100 m buffer + 1 month-cumulative rainfall + Rock cover + Slope + Season + Study area + (Year) + (ID plot)	8	-10872.46	0	0.398
Second best	Patch perimeter + Woodland percentage in a 1100 m buffer + 1 month-cumulative rainfall + Rock cover + Season + Study area + (Year) + (ID plot)	7	-10872.17	0.290	0.344
Third best	Patch perimeter + Woodland percentage in a 1100 m buffer + 1 month-cumulative rainfall + Slope + Season + Study area + (Year) + (ID plot)	7	-10871.59	0.866	0.258

Table S2. Parameters estimated by the global GLMM predicting wild boar proportion of rooted area in sampling plots: coefficients (B) and 95% confidence intervals (95% CIs). Variance of random intercepts is also shown. The reference category for 'Study area' is 'ALNR'. The reference category for 'Season' is 'Spring'. An asterisk marks the coefficients whose 95% CIs do not include 0.

**Response variable**	**Predictor**	**B coefficient**	**95% CI**
Proportion of rooting	Intercept	-3.345	-3.694; -2.996*
σ^2_{Year} = <0.001	Slope (%)	-0.114	-0.256; 0.027
$\sigma^2_{ID\ plot}$= 0.235	Patch perimeter (m)	0.185	0.048; 0.321*
	Woodland % in a 1100 buffer	0.247	0.048; 0.445*
	Rock cover (%)	-0.109	-0.229; 0.010
	Distance from persistent water	0.014	-0.094; 0.121
	One month-cumulative rainfall	0.210	0.128; 0.293*
	Study area (MRP)	0.451	0.012; 0.914*
	Study area (MPNR)	0.912	0.509; 1.315*
	Season (Summer)	-0.266	-0.434; -0.098*
	Season (Autumn)	-0.025	0.231; 0.181*
	Season (Winter)	0.301	0.128; 0.474*
	Soil depth class	0.027	-0.082; 0.136
